# Comparison of clinical features and outcomes between two age groups of cryptorchidism testicular torsion in children: a retrospective study in single center

**DOI:** 10.3389/fped.2024.1296222

**Published:** 2024-02-20

**Authors:** Shengxiong Wang, Haohui Tang, Jingmin Zhang, Ying Qiu, Xianghui Xie

**Affiliations:** ^1^ Department of Urology, Children’s Hospital, Capital Institute of Pediatric, Beijing, China; ^2^ Graduate School of Peking Union Medical College, Chinese Academy of Medical Sciences, Beijing, China; ^3^ Capital Institute of Pediatric, Beijing, China

**Keywords:** testicular torsion, cryptorchidism, orchidopexy, children, retrospective study

## Abstract

**Objective:**

The purpose of this study was to compare the clinical characteristics and outcomes of children with cryptorchidism testicular torsion between the younger age group and the older age group.

**Methods:**

We collected the clinical data of children with cryptorchidism complicated with testicular torsion in our hospital from January 1, 2013 to January 1, 2023. The patients were divided into two groups: the younger age group (1month∼4 years old, *n* = 7) and the older age group (4∼18 years old, *n* = 7). The differences of clinical manifestations and surgical results between the two groups were compared.

**Results:**

A total of 14 patients with unilateral cryptorchidism testicular torsion were included in this study, including 9 on the left side and 5 on the right side. The main clinical manifestations were pain /swelling of groin. The rate of crying in the younger age group was significantly higher than those in the older age group [(5,71.4%) vs. (0,0.0%), *P* < 0.05]. The median duration of symptoms of the younger group was less than the older group [42(7,96) h vs. 70(24, 96) h, *P* > 0.05]. The ipsilateral testicular salvage rate in the younger age group was 14.3% (1/7), which was lower than the older age group 57.1% [(4/7), *P* > 0.05]. The degree of testicular torsion in younger age group was more severe than the older age group [720(360, 1,080)° vs. 360(270, 360)°, *P* > 0.05].

**Conclusions:**

The overall salvage rate of cryptorchidism testicular torsion is low. Although the duration of symptoms in the older age group was longer, the salvage rate of the older age group seemed to be higher than that in the younger age group. In addition, physical and imaging examination of the reproductive system should be carried out in time to identify the children with cryptorchidism testicular torsion in the early stage.

## Introduction

Cryptorchidism is one of the most common congenital genitourinary disorders in pediatrics. Testicular torsion, inguinal hernia, testicular trauma, low fertility and testicular cancer are the complications of cryptorchidism (1, 2). Testicular torsion is a common emergency in pediatric urology. The incidence of testicular torsion is 3.8 per 100,000 males, and it is about ten times higher in patients with cryptorchidism (3, 4). Due to the high rate of testicular loss, accurate diagnosis and emergently medical treatments are often needed. The study found that the best outcome was achieved when patients were treated within 6 h from the beginning of the symptoms, up to 90% of the testes could be saved (5). After 24 h, the possibility of permanent ischemic damage or even necrosis of testis is greatly increased (6).

The most common symptom of children with testicular torsion is severe acute unilateral scrotal pain. Due to the typical clinical symptoms, testicular torsion is not difficult to diagnose. But in children with Cryptorchidism testicular torsion, symptoms are atypical. The main symptoms of them are crying, restless and vomiting, because the younger age children cannot describe their symptoms on their own. As a result, the opportunity for salvaging the testis is frequently missed. Moreover, study found that the testicular salvage rate was 16.7% (2/12) in the prepubertal patients, which was significantly lower than that in the post pubertal patients (60.6%, 6/10) (7). What's more, cryptorchidism and scrotal testicular torsion mainly occur in infancy and adolescence (8). Thus, we hypothesized that the clinical features and outcomes of children with cryptorchidism complicated by testicular torsion are different in two age groups.

## Materials and methods

After approving by the Ethics Committee of the Children's Hospital of Capital Institute of Pediatrics (SHERLL2022047), a retrospective review of data from patients with cryptorchidism complicated by testicular torsion was performed. Inclusion criteria were as follows: intraoperatively confirmed torsion of the undescended testis, follow-up for at least 6 months after surgery, and complete medical records. Unoperated and newborn patients were excluded. Using “testicular torsion” as the key word, we searched a total of 173 patients from January 1, 2013 to January 1, 2023 in the electronic medical record system in our hospital, and then with “cryptorchidism” as the key word, a total of 16 patients were retrieved. Two patients with incomplete clinical data were excluded, and 14 patients with cryptorchidism complicated by testicular torsion were finally included in this study. Two age groups were designed as the younger age group (1 month to 4 years) and the older age group (4∼18 years).

All patients were diagnosed as cryptorchidism testicular torsion by ultrasound and abdominal/inguinal surgical exploration. If ultrasound showed that there was no testicular sonogram in the scrotum, mass or edema echoes were detected in the ipsilateral groin or abdominal cavity, and the blood flow signal in the affected testes decreased or disappeared compared with the contralateral testes, emergency surgical exploration was carried out to conform the diagnosis. Additionally, either orchiopexy or orchidectomy was performed according to the vitality of the testis. If the testis became ruddy and flexible after manual manipulation, dartos pouch orchiopexy was performed. If the testis remained dark in color after wrapping it in warm saline-soaked towels for at least 5 min, the tunica albuginea was incised. If no new bleeding was identified after 15 min, orchidectomy was performed. Orchiopexy was performed on the contralateral testis of all patients at the same time. The patients were followed up in the outpatient clinic at 2 weeks, 1 month and 6 months after operation, and were examined once a year. Data were obtained by telephone follow-up, which were not recorded in detail on the medical records. Evaluation indicators were physical examinations and ultrasonography.Patients' clinical data were collected, including the age of onset, clinical symptoms, ultrasound results, duration of symptoms (defined as the interval between the onset of symptoms and the start of surgery), operation-related data and concomitant diseases. For those who could not describe the symptoms, their parents' description shall prevail. The outcome was the ipsilateral testicular salvage (ipsilateral testicular salvage was defined as testicular volume loss less than 50% of preoperative ipsilateral testicular volume after six-month operation) (9).

## Statistical analysis

Analyses were performed using SPSS, version 22.0 (IBM Corp., Armonk, NY, USA). Continuous variables were described as median and interquartile range (IQR) that did not comply with a normal distribution and were compared between two groups using the Mann-Whitney test. Qualitative or categorical variables were expressed as numbers and compared using the Fisher's exact test. All statistical tests were two-sided and performed with a significance level set at *P* < 0.05.

## Results

The patients' clinical features and Follow-up outcomes are summarized in Table 1. In our study, the prevalence of Cryptorchidism torsion was 9.25% (16/173) among all testicular torsion cases. A total of 14 patients with unilateral cryptorchidism testicular torsion were included in this study, including 9 on the left side and 5 on the right side. The median age in the younger age group was 10 (IQR: 6–14) months, while the median age in the older age group was 84 (IQR: 48–120) months. One patient had bilateral cryptorchidism and cerebral palsy in both group before surgery. Three patients were diagnosed as cryptorchidism torsion with vaginal process deformity in younger age group, the number of patients with that disease were more than the number in the older age group [(*n* = 3, 42.9%) vs. (*n* = 0, 0.0%), *P* = 0.096].

**Table 1:** Patients’ clinical features and follow-up outcomes (*n* = 14).

Variable	Younger age group (*n* = 7)	Older age group (*n* = 7)	*P*-Value
Age (month), median (IQR)	10 (6, 14)	84 (48, 120)	
Affected side, *n* (%)			0.500
Left	4 (57.1)	5 (71.4)	
Right	3 (42.9)	2 (28.6)	
Presenting symptoms, *n* (%)			
Inguinal mass and/or Inguinal pain	3 (42.9)	4 (57.1)	1.000
Abdominal pain	2 (28.6)	4 (57.1)	0.592
Restless	5 (71.4)	1 (14.3)	0.051
Limping	1 (14.3)	3 (42.9)	0.559
Vomiting	2 (28.6)	0 (0.0)	0.462
Scrotal swelling and/or empty	6 (85.7)	5 (71.4)	1.000
Crying	5 (71.4)	0 (0.0)	0.021
Duration of symptoms (h), median (IQR)	42 (7, 96)	70 (24, 96)	0.480
Misdiagnosis, *n* (%)	2 (28.6)	4 (57.1)	0.592
Torsion angle(°), median (IQR)	720 (360,1080)	360 (270,360)	0.104
Comorbidity			
Ipsilateral vaginal process deformity, *n* (%)	3 (42.9)	0 (0.0)	0.096
Cerebral palsy, *n* (%)	1 (14.3)	1 (14.3)	0.769
Recovery of testicular blood flow after torsion reduction, *n* (%)	3 (42.9)	5 (71.4)	0.296
Surgery, *n* (%)			0.296
Orchidopexy	3 (42.9)	5 (71.4)	
Orchiectomy	4 (57.1)	2 (28.6)	
Follow-up of children with orchidopexy			
Ipsilateral testicular salvage, *n* (%)	1 (14.3)	4 (57.1)	0.266
Follow-up time (month), median (IQR)	16 (8,20)	15 (10,18)	0.847

IQR, Interquartile range.

The common clinical manifestation of the two groups is scrotal swelling or pain in the inguinal region. Crying at presentation was significantly more prevalent in the younger age group (*n* = 5, 71.4%) than that in the older age group (*n* = 0,0.0%; *P* = 0.021). Restless in presentation were more prevalent in the younger age group than that in the older age group [(*n* = 5, 71.4%) vs. (*n* = 1, 14.3%), *P* = 0.051]. The median duration of symptoms of the younger age group was less than the older age group [42(7,96) h vs. 70(24, 96) h, *P* = 0.480], but the differences were not significant. Additionally, misdiagnosis was less in the younger age group (*n* = 2, 28.6%) than that in the older age group (*n* = 4, 57.1%; *P* = 0.592).

The degree of testicular torsion in younger age group was more severe than the older age group [720(360,1080)° vs. 360(270,360)°, *P* = 0.104]. Testicular blood flow was restored in 3(42.9%) patients in younger age group and it was 5(71.4%) patients in the older age group (*P* = 0.296). Although the testicular blood flow of case1, case 2 and case 6 were not satisfied, parents strongly demanded that the testes be retained. Therefore, orchidopexy was subsequently performed in 8 patients in total. Among the patients who underwent orchidopexy, there were 3 patients with postoperative testicular atrophy (postoperative testicular volume decreased by more than 50% compared with the preoperative), including 2 cases in the younger age group (case 1 and case 2) and one case in the older age group (case 6). (Table 2) The testicular salvage rate in the younger age group was 14.3% (1/7), which was lower than the older age group [57.1% (4/7); *P* = 0.266].

**Table 2:** Clinical features of 8 patients with orchidopexy and follow-up outcomes.

Patient	Affected side	Duration of symptoms	Torsion angle	Preoperative testicular size (cm^3^)	Testicular size at the last follow-up (cm^3^)	Contralateral testicular size at the last follow-up (cm^3^)	Follow-up
1^a^	right	96 h	720°	1.1 × 0.7 × 0.6	0.7 × 0.6 × 0.3	1.2 × 0.9 × 0.9	1 year
2^a^	left	42 h	1,080°	0.9 × 0.8 × 0.7	0.6 × 0.4 × 0.3	1.5 × 1.2 × 0.8	7 years
3^a^	right	22 h	180°	0.8 × 0.7 × 0.7	0.9 × 0.7 × 0.7	1.2 × 1.0 × 0.9	6 months
4^b^	right	10 h	270°	1.3 × 0.8 × 0.6	1.2 × 0.8 × 0.7	1.8 × 1.0 × 1.0	5 years
5^b^	left	96 h	270°	2.6 × 2.0 × 1.2	2.4 × 2.0 × 1.4	4.1 × 2.7 × 1.7	2 years
6^b^	left	168 h	180°	1.3 × 0.9 × 0.6	0.9 × 0.5 × 0.4	2.0 × 1.3 × 1.0	1 year
7^b^	left	24 h	360°	1.2 × 0.8 × 0.7	1.2 × 0.6 × 0.7	1.5 × 0.9 × 1.1	1.5 years
8^b^	left	60 h	360°	1.1 × 0.8 × 0.5	1.1 × 0.7 × 0.6	1.6 × 0.9 × 1.1	1 year

^a^ Younger age group.

^b^ Older age group.

## Discussion

To the best of our knowledge, there are little researches about cryptorchidism testicular torsion to compare between groups according to the high-risk age. Testicular torsion in patients with cryptorchidism accounts for 6.8%∼21.0% of all testicular torsion cases (10–13). In our research, the prevalence of cryptorchidism torsion is 9.25% (16/173), which is the same as other studies. Accordingly, We analyzed the clinical data of patients with cryptorchidism testicular torsion in two ages to supplementing more cases and providing valuable comparison from our center.

It is different that the early-stage symptoms of cryptorchidism testicular torsion in two age groups and it is difficult to differentiate cryptorchidism testicular torsion from acute abdomen by symptoms. The most typical clinical presentations in patients with cryptorchidism testicular torsion were inguinal pain/mass and an empty/swelling ipsilateral scrotum (14). However, the early-stage symptoms of cryptorchidism testicular torsion are not typical, usually abdominal/groin pain, nausea, vomiting and other symptoms of acute abdomen. Inguinal mass and scrotal swelling are the most common presentations happened in the late-stage (15). In our study, crying and restless at presentation are significantly more prevalent in the younger age group. Consequently, the younger age children were taken to see a doctor earlier than the older age children, and the duration of symptoms in the younger age children was shorter than the old one. In addition, 6 (42.9%) patients with early-stage symptoms of abdominal pain and vomiting were misdiagnosed as gastroenteritis and abdominal colic in the primary medical facilities, hence, those patients missed the best treatment time. Therefore, what we want to highlight is that the physical and imaging examination of the reproductive system should be carried out in time to identify the children with cryptorchidism testicular torsion in the early stage, especially with symptoms of crying, vomiting and abdominal/inguinal pain that cannot be explained by other reasons such as acute abdomen.

The overall salvage rate of cryptorchidism testicular torsion is low, but a higher salvage rate is more likely to be found in the older age children. Surgery within 6 h of normal testicular torsion is generally successful, but the success rate in cryptorchidism testicular torsion is below 40% due to often delayed diagnosis (16). The overall salvage rate of our center is 36% (5/14) which is the same as other researches. The older children with cryptorchidism testicular torsion seem to have a better prognosis than the toddlers. According to our data, the salvage rate of the older age group was higher than that in the younger age group, although the differences were not significant. We supposed that the degree of torsion in the older age group is lighter than that in the younger age group which account for a better salvage rate in the older age group. Thus, we believe that the cryptorchidism testicular torsion children in the older age group have a higher salvage rate than that in the younger ones.

The mechanism of cryptorchidism torsion remain unclear and patent vaginal process seems to be a risk factor for cryptorchidism torsion (8, 17). Cryptorchidism is prone to torsion due to the lack of anatomic fixation of the gonads in the scrotum and the possibility of spasmodic contractions of the cremaster muscle (18, 19). Further, polar attachment of gubernaculum and association of patent processus vaginalis also allow the testes a degree of freedom to rotate, predisposing to torsion (20). In our study, 3 out of 7 patients had patent vaginal process in the younger group, which appears to lead to increased testicular mobility and a greater likelihood of cryptorchidism torsion. That's maybe the reason why the degree of testicular torsion in younger group was more severe than older age group. Therefore, patent vaginal process seems to be a risk factor for cryptorchidism torsion. Besides, 2 cases with cerebral palsy eventually lost testicles even though one of them was treated in 17 h. For patients with cerebral palsy, missing the optimal operation time is more likely to lead to adverse consequences (21, 22).

## Limitations

The limitations of this study are as follows: (1) this study was a retrospective clinical analysis, which may leave out some cases that can not be found in the electronic medical record system; (2) due to the rarity of cryptorchidism testicular torsion, we got a small number of cases; (3) due to the ten-year time span of case collection, parents may have memory bias when we ask the reasons for the delay in our telephone follow-up.

## Conclusion

The overall salvage rate of cryptorchidism testicular torsion is low. Although the duration of symptoms in the younger age children was less than that in the older age children, the testicular salvage of the older age children is higher. Additionally, we want to highlight that physical and imaging examination of the reproductive system should be carried out in time to identify the children with cryptorchidism testicular torsion in the early stage, especially with symptoms of crying, vomiting and abdominal/inguinal pain.

## Data Availability

The raw data supporting the conclusions of this article will be made available by the authors, without undue reservation.
